# Evaluating the effect of a behavioural intervention bundle on antibiotic use, quality of care, and household transmission of resistant Enterobacteriaceae in intervention versus control clusters in rural Burkina Faso and DR Congo (CABU-EICO)

**DOI:** 10.1186/s13063-023-07856-2

**Published:** 2024-01-27

**Authors:** CABU-EICO consortium, Marianne van der Sande, Brecht Ingelbeen, Marie Meudec, Esther van Kleef, Linda Campbell, Edwin Wouters, Joachim Marien, Rianne van Vredendaal, Herwig Leirs, Daniel Valia, Sibidou Yougbare, Stephane Kouanda Juste, Aminata Welgo, Halidou Tinto, Delphin Mpanzu, Bijou Mbangi, Cesar-Augustin Khoso Muaka, Oscar Kiabanza, Adna Melanda, Richelin Makuaya, Didier Ndomba, Papa Mamadou Diagne, Leonard Heyerdahl, Tamara Giles-Vernick, Sandra Van Puyvelde, Ben Cooper

**Affiliations:** 1 Institute of Tropical Medicine (ITM), Antwerp, Belgium; 2 Universiteit Antwerpen, Antwerp, Belgium; 3 Clinical Research Unit of Nanoro (CRUN), Nanoro, Burkina Faso; 4 Centre de Recherche de Sante de Kimpese (CRSK), Kimpese, Democratic Republic of Congo; 5 Institut Pasteur, Paris, France; 6 University Cambridge, Cambridge, England; 7 University Oxford, Oxford, England; 8 Julius Center for Health Sciences and Primary Care, University Medical Centre Utrecht, Utrecht, Netherlands

**Keywords:** AMR, antibiotic use, WaSH, sub-Saharan Africa, participatory research

## Abstract

**Background:**

Antimicrobial resistance (AMR) is a rising threat in low-resource settings, largely driven by transmission in the community, outside health facilities. Inappropriate antibiotic use is one of the main modifiable drivers of AMR. Its risk is especially high in poor resource settings, with limited diagnostic and surveillance capacities, and many informal medicine vendors determining community use. We hypothesise that to optimise community antibiotic use, especially Watch antibiotics (recommended only as first-choice for more severe clinical presentations or for causative pathogens likely to be resistant to Access antibiotics), both the supply side (medicine vendors) and the demand side (communities) should be pro-actively involved in any intervention.

**Methods:**

In two existing demographic health surveillance sites (HDSS) in Burkina Faso and in the Democratic Republic of Congo, behavioural intervention bundles were co-created in a participatory approach, aiming to rationalise (Watch) antibiotic use and improve hygiene and sanitation practices. Bundles consisted of interactive interventions, including theatre, posters, discussions, etc. To evaluate impact, 11 of 22 clusters (a HDSS community with at least one (in)formal medicine vendor) were randomly assigned to this intervention, which will run over a year. The effect of the intervention will be evaluated by comparing outcomes before and after in intervention and control villages from a) exit interviews of clients from vendors, b) mystery patients presenting to vendors with a set of predefined symptoms, c) household interviews to assess behavioural changes related to antibiotic use, health literacy and water-sanitation-hygiene indicators. Long-term impact on AMR will be estimated by modelling changes in resistant Enterobacteriaceae carriage from repeated household surveys before, during and after the intervention in both arms.

**Discussion:**

Most existing interventions aimed at improving antibiotic use focus on health care use, but in resource-limited settings, community use is highly prevalent. Previous studies targeting only providers failed to show an effect on antibiotic use. Evaluation will be done with before-after epidemiological measurements of actual prescriptions and use. If effective in reducing (Watch) antibiotic use, this would be an empowering methodology for communities, which has significant promise for long-term impact.

**Trial registration:**

ClinicalTrials.govNCT05378880. 13 May 2022.

**Supplementary Information:**

The online version contains supplementary material available at 10.1186/s13063-023-07856-2.

## Background

Antimicrobial resistance (AMR) is a global health emergency, with a significant negative impact on health and socioeconomic development, in particular in low-resource settings. A major driver of AMR emergence is antibiotic use, where community-level antibiotic consumption, in addition to individual-level usage, has been associated with an increased risk of individual community members acquiring AMR bacteria [1]. As a result of difficult or delayed access to hospitals and formal health centres in many low-resource communities, self-medication with antibiotics from formal and informal medicine outlets, frequently without medically qualified staff, is widespread [2]. In sub-Saharan Africa, over two thirds of visits to community medicine stores may result in dispensing prescription-free antibiotics [3]. In the absence of a clinical or microbiological diagnosis, and with antibiotic sales a major source of revenue, community medicine outlets often dispense antibiotics without a clear rationale. Importantly, these antibiotic courses frequently consist of antibiotics classified by WHO as ‘Watch’ antibiotics, which should be restricted to specific indications due to their higher AMR potential [4, 5].

At the same time, healthcare providers or dispensers in medicine outlets may be faced with a strong demand from their communities for antibiotic treatments which, in the absence of a clinical assessment, risks to result in unnecessary or inappropriate antibiotic dispensing and usage. One RCT in India evaluated a bundle intervention targeting informal medicine outlets with an intensive multi-topic training programme over 9 months and found it improved correct clinical case management [6]. Unfortunately, a direct effect on the frequency of antibiotic use could not be demonstrated. The intervention, however, only targeted informal medicine outlets and did not seek to address the demand for antibiotics from the community.

To modify community antibiotic use and thus limit the spread of AMR, it is key to understand what factors can induce and sustain the necessary behavioural changes. At the same time, to reduce transmission of bacteria and AMR genes, as well as the burden of infectious diseases triggering antibiotic demand, strengthening of prudent WaSH practices is needed, also taking into account that in rural sub-Sahara Africa people and animals live in very close proximity. As defined by the COM-B model, behaviour is shaped by three essential conditions: capability, opportunity, and motivation, which provide intervention opportunities for behaviour change. Capabilities include psychological (e.g. knowledge) and physical (e.g. skills) capabilities. Opportunity includes social and physical opportunities (e.g. social influences and environmental context and resources). Motivation includes reflective and automatic motivation such as beliefs about capabilities and consequences, goals, and ideas about professional role and identity [7]. Target behaviours for interventions are to be based upon considerations around impact potential, likelihood for change, potential spill-over effects as well as ease of measurement.

Thus, a multifactorial approach is needed, actively involving both formal and informal healthcare providers as well as the population in the design and implementation of such stewardship interventions for a sustainable impact. This article describes the trial design by which the effect of this behavioural intervention will be evaluated on (Watch) antibiotic use, on clinical care and on water-sanitation-hygiene (WaSH) behaviour. For this, we adhered to the SPIRIT reporting guidelines [8] (Annex 1).

## Methods

### Study design

In a cluster randomised trial, we will evaluate if a year-long behavioural intervention bundle in a rural setting in Burkina Faso (Nanoro district) and in the Democratic Republic of Congo (DRC) (Kimpese district) has a superior outcome on a range of standardised AMR and WaSH indicators in intervention and control villages. Controlled measurements of antibiotic use (patient surveys), patient management (mystery patients) and water-sanitation-hygiene (household surveys) will be collected in the same season before and after. In each setting, eligible villages and town neighbourhoods (clusters) will be randomly allocated to intervention or no intervention (control). Randomisation is in two strata per site. In Nanoro, strata distinguished villages with primary health centre and those without (only formal or informal medicine outlets). In Kimpese, strata distinguished peri-urban neighbourhoods of rural villages. For each stratum, clusters (villages or neighbourhoods) are randomised 1:1 as intervention or control. A random number between 0 and 1 is given to each cluster using the RAND() function in Excel by a local data manager not involved in the trial. Half of the cluster in each of both strata, those with the higher numbers, are selected as intervention clusters; the other half, with the lower numbers, as control clusters. Allocation of villages to intervention or control was concealed until all had been recruited and baseline data available.

By randomising communities rather than individuals, contamination of intervention and no intervention should be maximally avoided, as supported by pilot studies in the DRC that observed that >80% of social contacts were with people in the same village or neighbourhood (manuscript in preparation). A steering committee consisting of the PIs of all 7 participating institutes will meet at least annually to discuss progress, and at least annually full consortium meetings will be organised with options for on-line participation.

### Study setting and population

Health demographic surveillance sites (HDSS) exist in Nanoro district, Burkina Faso, and Kimpese district, DRC where trusted long-term contacts have been built with the communities. This provides a fertile environment for high-quality studies [9]. The HDSS in Nanoro was established in 2009 and currently includes a population of about 60,000 people, living in 24 villages. 16 of these villages have a primary care health facility, to which a community pharmacy store is linked. The HDSS in Kimpese was established in 2018 and included a population of about 170,000. In the central town of each HDSS (Nanoro and Kimpese), there is a public district referral hospital, with a clinical and a research laboratory attached. Both HDSS have quality-controlled bacterial culture and antibiotic susceptibility testing capacity installed at the district hospital level. Experienced social science teams are integrated in both HDSS.

Populations in these sites are well-characterised in terms of demographics, socio-economic status, healthcare utilisation and access-to-water and hygiene parameters. Essential demographic information is updated on a continuous basis through village-based reporters, while structural information is updated during annual rounds, as well as during dedicated study visits. Baseline demographic and health indicators of households are collected at 4–6 monthly intervals, and clinical microbiology surveillance has been established in the district referral hospitals [10, 11].

Pilot studies in the HDSS of both settings, confirmed that health centres (66% in Nanoro, 68% in Kimpese), private clinics (none in Nanoro, 4% in Kimpese), community pharmacies (17% in Nanoro) and informal medicine vendors (16% in Nanoro, pharmacies and informal vendors 18% in Kimpese) were the main sources of antibiotics [12, 13]. Frequent consumption of non-prescribed antibiotics was reported by community members exiting from these local medicine stores, with a significant proportion consisting of Watch antibiotics (20% in Nanoro [14] and 24% in Kimpese [13]). Furthermore, purchased antibiotic courses were frequently underdosed or interrupted [13, 14].

Implementing the behavioural intervention bundle will not require alteration to usual care pathways and these will continue for both trial arms.

### Theoretical Framework of the Intervention

The COM-B model for behaviour change will underpin the development of the intervention [15]. Widely used in designing interventions [16], COM-B identifies three essential conditions for behaviour — capability, opportunity, motivation: Capabilities include psychological (e.g. knowledge) and physical (e.g. skills) capabilities; Opportunity includes social and physical opportunities (e.g. social influences and environmental context and resources). Motivation includes reflective and automatic motivation such as beliefs about capabilities and consequences, goals, and ideas about professional role and identity [7].

### Intervention development and implementation

**Fig. 1:**
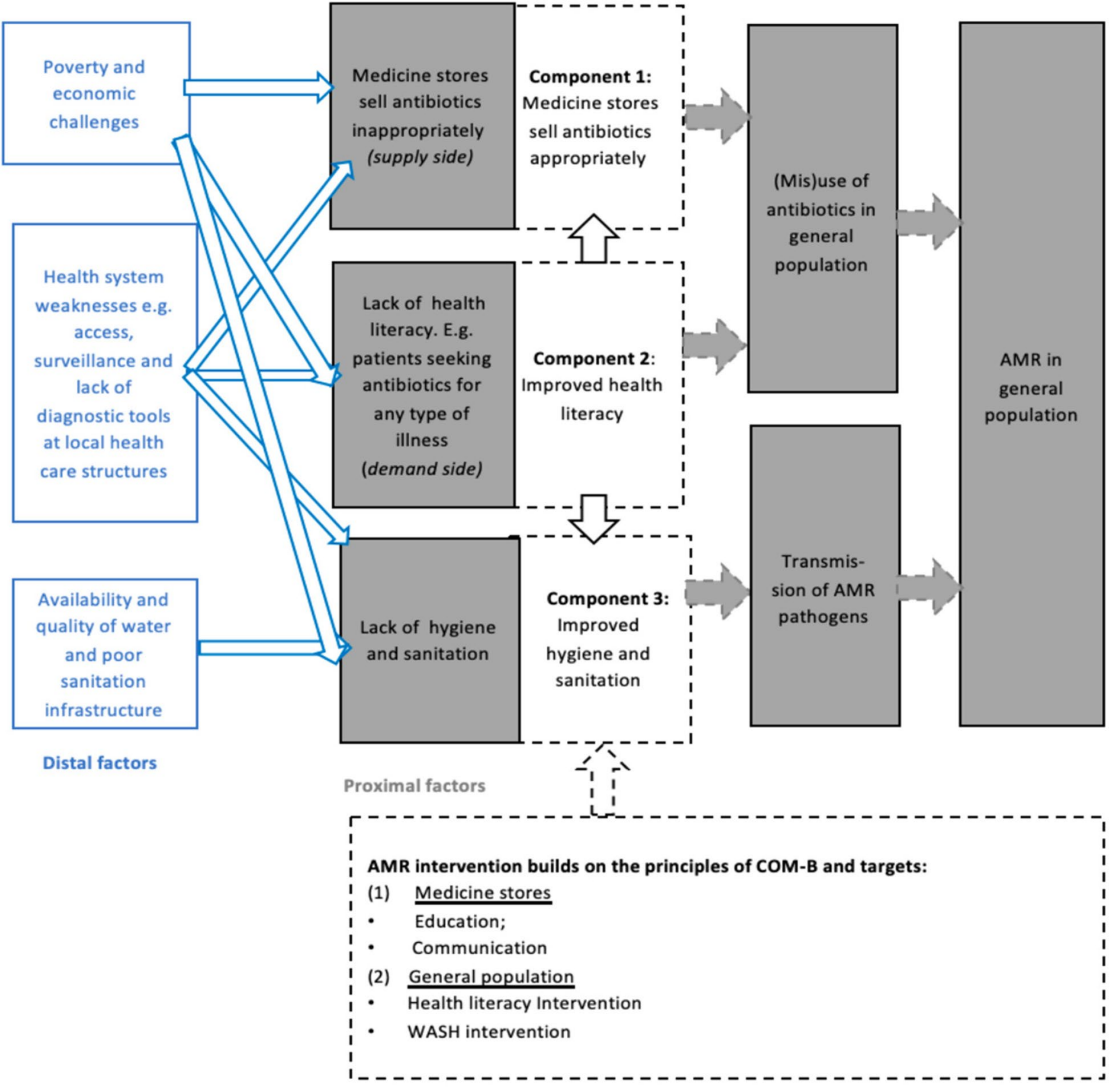
Drivers of AMR, guiding the development of the behavioural intervention

Each intervention cluster will be visited 3 times over the period of a year, for one week at a time. The intervention bundle targets behaviours which are shaped by identified drivers of AMR. These drivers either affect the selective pressure by antibiotics on bacteria, or the transmission of AMR bacteria. At the community level in low-income settings such as Nanoro or Kimpese, drivers of AMR include suboptimal health literacy of the population, suboptimal selling practices of antibiotics in medicine stores, limited timely access to formal healthcare, poor formal healthcare provisioning of diagnostic tools and services, and widespread incidence of infections related to poor hygiene and sanitation (Fig. 1).

Intervention development during the initial 8 months of the project followed the gold standard Intervention Mapping framework, with 6 crucial steps for intervention development [17, 18]. A preparatory phase, utilising Photovoice methodology [19], focus group discussions and individual interviews, explored determinants of antibiotic misuse and drivers and barriers to change. Analysis of the data gathered during the preparatory stage resulted in the participatory co-creation of the intervention bundles (for example, many communities suggested theatre as an effective form of generating discussion), as well as the most appropriate angles for discussion: for example, targeting local vendors in DRC using the idea of their vital signposting role, and emphasising their importance in safeguarding the health of the community.

A detailed context-specific day-to-day description of activities with the communities (adults, adolescents, schools) and with the formal and informal providers was subsequently developed for each HDSS.

The first component of the intervention bundle targets medicine stores, incorporating a series of individual and group sessions with both formally trained community pharmacy staff as well as informal vendors of antibiotics focusing on when antibiotics should and should not be used, as well as communication skills with customers.

The second component of the intervention bundle targets the general population and intends to increase community health literacy around health care use and communication with health care providers. Health literacy is a key concept in reducing incorrect antibiotic use, entailing “knowledge, motivation and competences to access, understand, appraise, and apply health information in order to make judgements and take decisions in everyday life concerning healthcare, disease prevention and health promotion to maintain or improve quality of life during the life course.” [20].

The third component of the intervention bundle targets the general population, focusing on improving water and hygiene and sanitation practices in order to prevent infectious illness and the (perceived) need for antibiotics. Activities determined upon by the teams include discussions with village and religious leaders, theatre and films, school games, and ad-hoc sessions. The activities used for these components draw on the ideas around hygiene and disgust [21, 22], activities from the World Bank’s Handwashing Handbook and successful WASH interventions such as SuperAmma [23]: the ADAPT guidelines were followed in the adaptation of certain activities from SuperAmma [24].

There will be no special criteria for discontinuing or modifying allocated interventions.

### Timeline

**Fig. 2:**
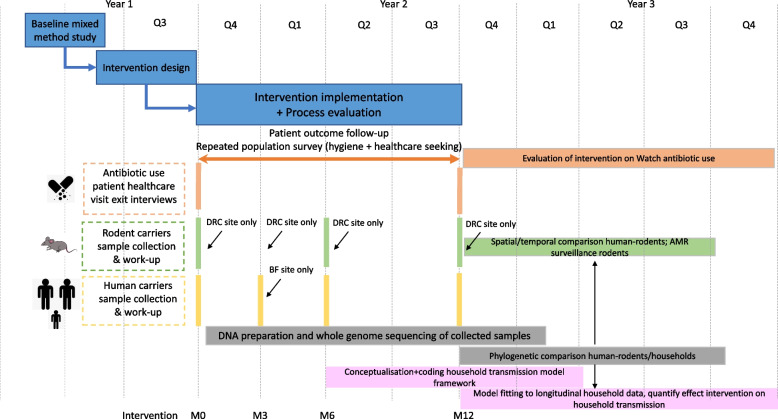
Timeline. (Legend: DRC, Democratic Republic of Congo; BF, Burkina Faso)

The timeline is shown in Fig. 2.

### Objectives

#### Primary objectives

1. Develop, implement, and evaluate the effect of a behavioural intervention bundle targeting medicine stores (including community pharmacies and informal medicine sellers), and the surrounding populations on (Watch) antibiotic use.
2. Develop and pilot environmental AMR surveillance through rodent surveillance.
3. Estimate and model the effect of the intervention bundle on AMR prevalence and transmission, focusing on faecal E. coli and Salmonella carriage.

#### Secondary objectives

1. Estimate the intervention bundle’s effect on hygiene, on case management by medicine stores and on clinical outcomes.
2. Identify pathways and incentives through which educational or peer influence interventions improve quality of care.
3. Compare prevalence of AMR bacterial populations in human (i.e. household members) and rodent reservoirs; compare with AMR prevalence in routine BSI surveillance.
4. Spatial and ecological analysis as well as phylogenetic comparison of AMR bacterial populations and genetic clones identified in human carriers and dwelling rodents.
5. Quantify household transmission of AMR genes or pathogens and estimate the relative importance of an environmental transmission source.

### Outcomes

#### Primary outcome

1. Changes in use of (Watch) antibiotics from health centres and medicine stores.
2. Estimated changes in AMR community carriage.

#### Secondary outcomes

1. Assessment of impact of educational or peer influence interventions on improved antibiotic use, quality of care, health literacy and WaSH practices.
2. Estimate households’ risk of community-level AMR transmission, including WaSH conditions and behaviour, contact with domestic animals.
3. Changes in correct patient management, estimated from simulated patient visits, during which so-called mystery patients present with well-defined and common infectious disease-related clinical presentations.

### Eligibility

All villages or neighbourhoods with at least one health centre or medicine outlet and at least 500 inhabitants are eligible to be recruited as study cluster. If more such villages are present, the villages with the larger number of medicine outlets will be selected. Medicine outlets take different forms, going from community pharmacies with medicine dispensed by staff with various qualifications, to medicine shops owned by qualified healthcare workers but with actual medicines dispensed by lay staff, to fully informal medicine vending in regular shops or on markets. All community members or providers present at the time of assessments or interventions were invited to participate. The unit of randomisation corresponds to a village with at least one medicine store.

### Sample size

The sample size is based on the primary outcome. Including in each setting 22 clusters, with per cluster 100 patients to be interviewed upon exiting a medicine store, a frequency of Watch antibiotic use of 24% in DRC [13], and assuming an estimated intraclass coefficient of 0.05, a cluster level correlation (rho_c_) of 0 (conservative), subject level correlation (rho_s_) of 0 (different subjects at baseline and at 12 months), our study has 80% power to observe a 40% reduction in Watch antibiotic use.

### Data collection

Data collection will be done twice: first pre-intervention, and repeated a year later, post-intervention, to account for seasonality in antibiotic use, health care seeking behaviour and in WaSH practices (see also timeline, Fig. 2):

1. Repeated patient visit exit surveys: After completing a healthcare visit, patients and parents of paediatric patients (regardless of whether they received an antibiotic) will be surveyed, using a structured questionnaire, on: symptoms, quantity of antibiotics dispensed/purchased by group of antibiotics if any, dose and duration of antibiotic treatment (including potential up/downscaling), mode of administration, number of antibiotics and antimalarials used concomitantly (including reasons). These surveys will be carried out pre- and post-intervention at identified medicine stores in intervention and control clusters.
2. Repeated visits by simulated patients are the gold standard to evaluate patient management and the appropriateness of antibiotic dispensing in medicine outlets: they present at the medicine outlet and answer to questions in a standardised way (trained before the visit) to mimic clearly outspoken medical conditions for which the indication to treat or refer cannot be debated [25]. Simulated patients are blinded whether the outlet they visit received intervention input or not. Visits are unannounced to avoid that providers adapt their behaviour during those visits to be more desirable when observed, a so-called Hawthorne effect. The most suitable tracer conditions will be identified during the formative research stage [6]. Case management is evaluated using a standardised scoring grid evaluating clinical assessment and anamnesis, actions taken and medicine dispensing during or following the visit
3. Repeated household surveys: during repeated household visits, pre- and post-intervention, we will record potential factors associated with AMR transmission (in particular, hygiene and infection control practices). Stool samples will be collected from a selected number of household participants for analysis of the presence of Enterobacteriaceae and their antibiotic susceptibility.
4. Rodents are an abundant and diverse group of small mammals, with several species living in close contact with humans and livestock, making them a relevant group for environmental surveillance of AMR [26]. To assess their potential in environmental AMR surveillance, rodents will be collected for stool sampling for similar analysis of the presence of Enterobacteriaceae and their antibiotic susceptibility twice, once during the dry season, once during the wet season, at different distances from human habitation, with a focus on the peridomestic species that are common in the study area (*Mus musculus*, *Mastomys natalensis*).
5. Following the implementation of the interventions, a qualitative process evaluation among providers and community members is planned to understand perceptions and intended and potentially unintended impact of the evaluation in a selection of the intervention clusters using focus groups and individual interviews with key stakeholders.

### Analysis

The effect evaluation will use standard experimental methods and will make use of multilevel statistical models (to account for clustering within villages and households), including time*intervention interactions. The population’s socio-demographics, socio-economic status, healthcare use and health behaviour are regularly recorded during scheduled HDSS rounds, which will allow subgroup analyses of the effect of the intervention.

Based on the exit interviews, the proportion of primary care or medicine outlet visits resulting in dispensing of Watch antibiotic courses will be estimated, adjusted for clustering, in the intervention and in the control groups before and 12 months after the start of the intervention. Antibiotics will be grouped as Access, Watch or Reserve (AWaRe) according to WHO’s 2019 AWaRe classification. Patients’ antibiotic treatment dose, dosage, duration, mode of administration and uptake will be assessed, to determine the Defined Daily Doses (DDD) each treatment course accounts for. We assess for each treatment course whether the treatment is underdosed, based on the 2022 WHO AWaRe Antibiotic Book. A case management score is deducted using the scoring grid to be completed after each simulated patient visit. The change in frequency of Watch antibiotic use and in mean case management score before and after the intervention (months 0 and 12) will be compared to that during the same period in the control areas. We will determine risk differences and risk ratios, which will be adjusted for socio-economic indicators and other potential confounding factors. Linear and non-linear regression models will be used to estimate the effect and carry out subgroup analyses.

Stool samples will be cultured on selective CHROMagar™ plates and species identified. If *E. coli* and/or *Salmonella* isolates are detected, they will be typed and antibiotic susceptibility testing done according to CLSI guidelines for pathogen-antibiotic indicators and for ESBL, if the minimal inhibitory concentration for ceftriaxone or ceftazidime is above 1 mg/L.

A selection of ESBL-producing *E. coli* isolates will be sequenced. DNA will be extracted and prepared for whole genome sequencing (WGS) using Illumina technology available through the Cancer Research UK facility. The WGS data will be bioinformatically assessed for quality and analysed to confirm species and sequence type per isolates. Furthermore, the genetic content of AMR genes and the presence of plasmid replicons will be determined. In-depth comparative genomics approach will be used to assess relatedness between isolates through reconstruction of the maximum likelihood phylogenies per species/sequence type, with their representative metadata including AMR data. From this, we can assess whether isolates belong to different clades or whether the isolates are interspersed, suggesting ongoing transmission between reservoirs. We will use a Bayesian approach (using BEAST [27]) to estimate transmission events.

We will also use these data to fit Markov models to longitudinal household data derived from repeated stool samples. This will enable estimation of the rate of person-to-person transmission within households, the rate of loss of detectable carriage within individuals, and the association of these rates with the study intervention and baseline household covariates. We will perform this analysis both at the level of the AMR gene and, where typing data permits, at higher levels. Individuals will be classified as non-carriers or carriers of AMR pathogens or genes, and intervention exposure is assumed to alter transition probabilities between states. Based on the results of the phylogenetic comparison of AMR bacterial populations, we will explore different hypotheses related to the relative importance of an environmental acquisition source, by considering different model structures allowing for the presence or absence of environmental acquisition.

Qualitative data will be analysed using NVivo following transcription of focus groups and individual interviews to identify dominant themes and explore perceptions.

No specific analysis is foreseen related to non-adherence or missing data as the intervention is at community level, as well as collection of pre and postintervention data. A community cannot not adhere or go missing; the intervention and data collection before and after will target those community members and providers who are present and willing to participate.

### Data management

A full data management plan has been drafted for the project in line with the requirements of the funders, and agreed upon by all partners, detailing all important aspects with regard to data management, data protection and open access to data. European researchers subscribe to the European Code of Conduct for Research Integrity. These policies ensure data integrity, traceability and confidentiality. Project data will be shared between consortium members on a need-to-know basis. Since personal data is collected and processed, the project will comply with the EU General Data Protection Regulation (GDPR 2016/679). Anonymisation or at least pseudonymisation will be done at the earliest opportunity and as much as possible. No participant-identifiable data will be disclosed from study intervention sites/partners, nor in study databases, analysis and reports. User roles and access controls for study-sensitive data will be implemented. Controlled access to confidential data can be granted on a need-to-know basis, involving a local data access committee. Data storage will be done on centrally secured and protected servers or clouds, with secure (encrypted) transfers of study confidential data if needed. Data retention periods will be in line with legal and data protection requirements but will be at least 5 years after completion of the research.

### Dissemination

Results will be shared with the local HDSS and health authorities in interactive meetings and with posters. National and international authorities will be informed with policies briefs. Press releases will encourage the regular media to include information for the general public in their publications. Scientific results will be disseminated via peer-reviewed and (in principle) open-access publications in non-predatory journals, and through presentations in AMR networks. Pseudonymised data will be open access on a study repository, following the publication of results.

### Ethical approval

Ethical approval for the final protocol vs 1 has been obtained from the Institutional Review Board of the Institute of Tropical Medicine, Antwerp, Belgium (1559/22, dd 29032022; 1566/22, dd 24052022), from the Ethics Committee of the Antwerp University Hospital, Belgium (3363, dd09052022; 3456, dd 24052022), from the Ethics Committee for Health Research in Burkina Faso (2022-03-050, dd 02032022), and from the Ethics Committee of the Université Protestante au Congo, DRC (CEUPC0098, dd 31032022).

Informed consent for the intervention is collected orally from village chiefs and documented, and written consent if obtained from participating health care providers or medicine dispensers. For patients’ participation to the exit surveys and for household heads’ participation to the household surveys, written informed consent is obtained.

A risk management plan has been included in the proposal.

In case of protocol amendments, following ethical approval, funders will be informed, and the revised protocol will be updated in the clinical trial registry. Any deviations from the protocol will be fully documented using a breach report form.

A data Monitoring Committee was not considered indicated as this behavioural intervention with no individual interventions or follow-up, and with data collection only at baseline data before the start of the trial and upon completion of the trial. Interim analysis or stopping rules therefore also do not apply.

### Funding

The study is funded under the EU Joint Programme Initiative (JPI) AMR Harissa call “One Health interventions to prevent or reduce the development and transmission of antimicrobial resistance” (JPI-AMRCalls@agencerecherche.fr). In agreement with JPI regulations, member state co-funding was provided by the Belgium FWO (to ITM and UA), the French ANSR (to IP), the UK MRC (to Oxford and Cambridge) and the Swedish SIDA (to CRUN and CRSK). General support to the HDSS in both countries was financially supported by the Belgium Development Cooperation.

The funders have no role in any phase of the study, including decisions related to publications or dissemination.

## Discussion

To combat the complex drivers of AMR, in line with recommendations from WHO and the World Organisation of Animal Health (OIA), a context-specific approach is needed which both promotes rational use of antibiotics, and optimal prevention of transmission. Our project proposes a robust participatory-driven behaviour change intervention to reduce the need and use of (Watch) antibiotics from formal and informal medicine stores, targeting both the demand (community) and supply (medicine store) side, to improve the quality of care of frequent clinical presentations from infectious disease, and to reduce emergence and transmission of AMR. A process evaluation after the second and third rounds of the intervention is therefore planned in a number of intervention villages in each country to better understand the contribution of each element of the intervention to intervention impact, as well as the processes underlying intervention impact.

The epidemiological before-after evaluation in intervention and control villages as described will assess the impact of this year-long intervention on actual antibiotic use, self-reported WaSH and health literacy changes, and clinical care by the medicine stores. The projected long-term impact of the intervention bundle on AMR transmission will be estimated via mathematical modelling, combining these outcomes with estimates of within-household AMR pathogen or gene transmission rates, estimates of AMR prevalence, and estimated environmental exposure. We will fit Markov models to longitudinal household data derived from repeated stool samples which will enable estimation of the rate of person-to-person transmission within households, the rate of loss of detectable carriage within individuals, and the association of these rates with the study intervention and baseline household covariates.

At the same time, it will be key to develop sustainable One Health surveillance to keep track of AMR changes over time and place, as these might signal the need for modification of control activities. In a sub-study in DRC, we will already pilot environmental surveillance with rodents (as sewage surveillance is not an option) in parallel to the community sampling, and we are planning to evaluate the possibilities of pooled metagenomic One Health surveillance of different clinical sources and potential environmental reservoirs, to monitor trends and predict clinical susceptibilities and AMR.

### Limitations

There are a number of limitations to this study. If proven effective, it is quite human resource intensive, as most complex interventions, which could raise some concerns about sustainability and scalability. Nevertheless, the cost of unrestricted further increases in AMR will far outweigh those of preventive interventions. Furthermore, we are convinced that the only sustainable way to change behaviour is by a joint, bottom-up process to have a common understanding with all stakeholders of problems and solutions. For sustained impact, it is likely indicated to have regular refresher meetings to maintain motivation, in view of many other daily life challenges faced by people in low-resource settings. By involving policy makers from the start, we hope to ensure they will be motivated to organise their preventive services in such a way as to enable this. However, there is always the risk that the prevention paradox means that policy makers are less inclined to invest in problems that are not directly visible. Follow-up studies should therefore address the cost-effectiveness of community-based interventions, and at the same time explore to what extent they could be supported by other community-driven activities, e.g. strengthened One Health surveillance at the community level, which is possible with the pooling of samples.

Furthermore, although we intended to demonstrate the added value of this innovative approach, we realise that the developed intervention itself is not generalisable nor reproducible as such by design. It requires co-creation and context-specificity to achieve the buy-in of participants and the appropriateness of the intervention elements aimed to nudge towards appropriate use of antibiotics and improved infection control practices.

Additionally, patient exit surveys are the most suited method to measure the prevalence and quantify of antibiotic use during visits to medicine stores across different provider types, yet an assessment of the appropriateness of antibiotic use is impossible in the absence of a detailed clinical assessment with extensive diagnostic tools. Moreover, antibiotic dispensing might be different -more desirable - among patients visiting the provider at the time an interviewer is present. Similarly, answers to household surveys might reflect what is considered more desirable, compared to day-to-day behaviour. We addressed this by adding to the questionnaire as much as possible observations by the interviewer. We will assess the risk of bias by comparing changes in reported behaviour to observed behaviour change. Finally, simulated patient visits are limited by the number of clinical presentations they can assess, in turn, limited to presentations that would not require invasive procedures and that are acceptable and feasible.

### Trial status

**Fig. 3:**
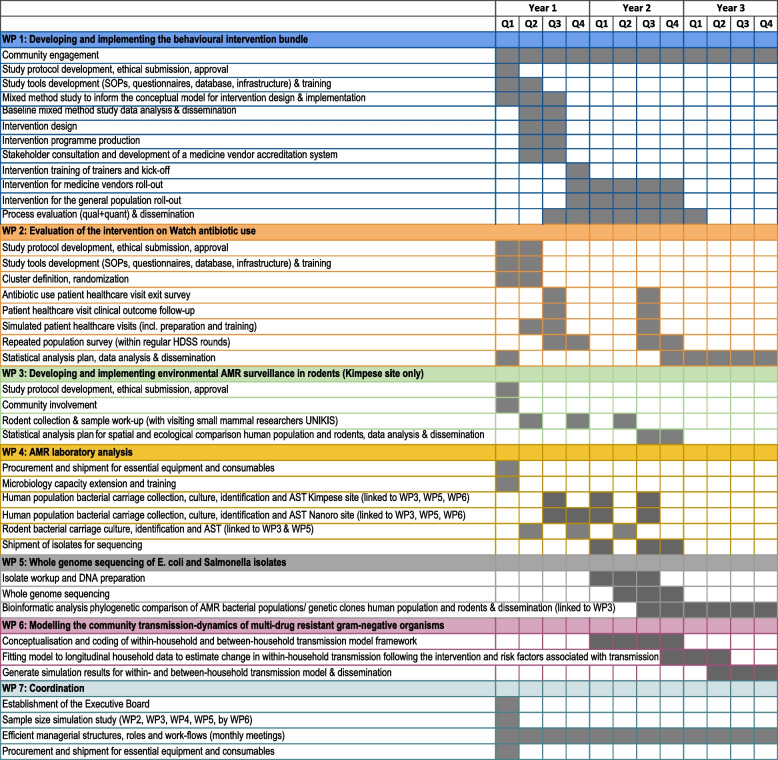
Workplan

**Fig. 4:**
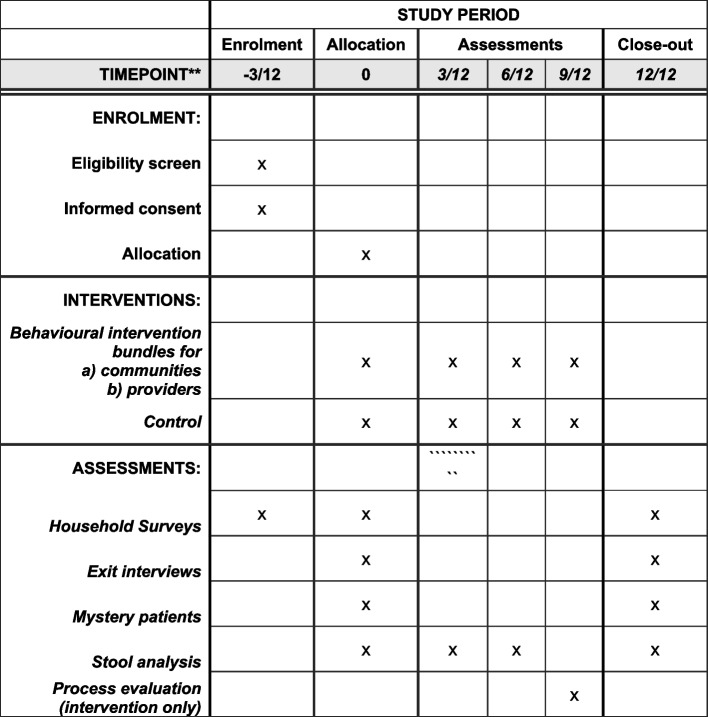
Spirit-figure CABU-EICO with schedule of enrolment, interventions, and assessments. See also Fig. 3 for details

Following external peer review, the study protocol was selected by JPI for funding on 28 October 2021 (JPIAMR2021-053) with a total budget of 1.6 million Euro, and registered at www.clinicaltrials,gov on 13 May 2022 (NCT05378880). In 2022, the development of the intervention bundles and materials took place through a co-creation process with the communities, per country 22 clusters were identified, and pre-trial data collection started in both arms. Implementation of intervention bundles started in February 2023 and is ongoing, with post-intervention bundle data collection in both intervention and control villages expected to be completed by summer 2024 (Figs. 3 and 4).

### Supplementary Information

**Additional file 1.** Spirit checklist.

**Additional file 2.** Informed consent forms.

**Additional file 3.** Biological specimens statement.

## Abbreviations

AMR: Antimicrobial resistance
AWaRe: Access-Watch-Reserve antibiotics (WHO classification)
BF: Burkina Faso
DDD: Defined daily dosis
DRC: Democratic Republic of Congo
ESBL: Extended spectrum beta lactamase
HDSS: Health Demographic Surveillance Site
JPI: Joint Programme Initiative
LMIC: Low and middle-income country
OIA: Organisation Animal Health
sSA: Sub-Saharan Africa
WaSH: Water, Sanitation, Hygiene
WHO: World Health Organisation
