# Supplementary material for: Evaluating the effect of a behavioural intervention bundle on antibiotic use, quality of care, and household transmission of resistant Enterobacteriaceae in intervention versus control clusters in rural Burkina Faso and DR Congo (CABU-EICO)
Source: Trials. 2024 Jan 27;25:91. doi: 10.1186/s13063-023-07856-2 (PMC10821568; doi:10.1186/s13063-023-07856-2)
Supplement: Supplementary file 2 — Additional file 2. Informed consent forms. [file 13063_2023_7856_MOESM2_ESM.docx]

| INFORMATION FORM FOR INFORMAL MEDICINE PROVIDERS |
| --- |

You are invited to voluntarily participate in a study conducted by the Clinical Research Unit of Nanoro (CRUN), the Institute of Tropical Medicine, Antwerp in Belgium, the University of Cambridge and the University of Oxford in the United Kingdom. The study and the intervention it will evaluate are described in this information sheet. If you have any questions about the study or intervention, please don’t hesitate to ask. In addition, you are of course free to interrupt your participation in the study or intervention at any time.

Purpose and description of the study

The increase in antibiotic resistance makes drugs currently used for bacterial infections ineffective. Before developing and introducing interventions that would enable better use of antibiotics in the community and prevent infections with resistant bacteria, we should first understand where, when, how and why antibiotics are used and under what conditions and practices of hygiene and sanitation pathogens transfer are facilitate between people. This is why this study is proposed.

From a consultation of the stakeholders involved, the aim is to develop a package of interventions (one for medicines providers and one for the entire population) that responds to the risk factors of antibiotic resistance, in order to improve the quality of care and meet the perceived needs of health professionals or the population.

Interventions and surveys will be implemented in 11 communities, and surveys will also be carried out in 11 other control communities – but without interventions. These surveys are:

- **Patients visit exit interviews** will be carried out at the end of a visit to a formal medicine provider before and one year after the intervention to measure a possible change in the use of antibiotics,
- **Simulated patients visits** will be carried out before and one year after the intervention to evaluate a possible change in the quality of care: actors will present themselves as patients with 5 different clinical presentations, and healthcare will be evaluated from a check list.

You will be asked to participate in the development of interventions, and - if you are a medicine dispenser in the study area - your agreement for your patients to participate in surveys on antibiotic use. You will be then be visited by stakeholders and thus the quality of care will be evaluated.

Time required for participation

You will be asked to set aside approximately one hour for an initial interview. A second one-hour session will be planned with some participants from the first session, for feedback on the proposed interventions.

The intervention will be implemented during one year, and will be offered in the form of training, feedback sessions, clinical discussions between peers (therefore between health actors) according to identified needs.

The intervention evaluation surveys will not require dedicated time from health actors.

Voluntary participation

Your participation in this study is entirely voluntary and this choice is not documented or reported in the study materials.

Don't hesitate to ask if there is anything you don't understand or ask for explanations until you are satisfied. You are free to withdraw or stop your participation at any time.

If you wish to participate, indicate that you agree to participate in the study, verbally, with a signature for this verbal consent by the researcher/investigator.

Privacy

All information collected about you during the study will be kept strictly confidential. Your name is not recorded and any information you provide will be kept confidential. Only two researchers from the research team will have access to the data and information collected. It is possible that an excerpt from the interview will be used in the dissemination of our results, but we will ensure that any information identifying you is removed from the quote.

Under no circumstances will your identity or contact details be shared with authorities or health authorities. We guarantee that all information will be treated strictly anonymously, and your name will be replaced by a code in all official study documents.

In the event of a group discussion, we ask you to respect the other participants (their anonymity) as well as the confidential nature of the conversations that will take place. However, if you are uncomfortable participating in a group conversation/discussion, we can answer your questions to reassure you. However, if you wish, you can cancel your participation.

Once you have confirmed your participation, we would like to record the audio of our conversation for research purposes only. You are free to refuse it, however, it will help our analysis and our work. All audio-recorded information will be destroyed after the study.

Benefits

There is no direct personal benefit to you from agreeing to participate in this study. However, consultation gives you the opportunity to propose interventions that can contribute to better public health in the community. Medicine dispensers will be able to participate in educational activities or scientific support, such as training and treatment tools, in order to optimize the use of antibiotics and thus improve patient care. Those who will be in the intervention communities will receive them for one year, the others after the year of evaluation of the intervention package. There is no financial compensation for participating in this study. If you had to travel, transport costs will be reimbursed (a lump sum).

Ethics committee

This study was reviewed by the institutional review board of the Institute of Tropical Medicine, the ethics committee of the University of Antwerp, Belgium, and the National Ethics Committee for Health Research in Burkina Faso, and received a favorable recommendation.

If you have any questions or comments about the study now, during or after your participation, you can contact the study coordinator: VALIA Daniel; Phone: +226 70285553; Email: valiadaniel@yahoo.com

-----------------------

| VERBAL INFORM CONSENT FORM FOR INFORMAL MEDICINE PROVIDERS  (TO BE COMPLETED BY THE RESEARCHER FOR EACH PARTICIPANT) |
| --- |

- I confirm that the information sheet has been read and explained to the respondent in a language that the respondent understands well.
- I ensured that the respondent understood that he/she can freely choose to participate in the study, that he/she does not have to answer a question that he/she prefers not to answer, and that he/she can end the interview at any time.
- The respondent had the opportunity to ask questions about the study and its objectives. If the respondent had questions, I or a colleague from the research team answered the questions correctly and clearly.
- The participant was explained that their name is not recorded except on the present form, which will be kept secure, only used if the participant would prefer to cancel their participation in the study, and destroyed one year after the end of the study. The information provided will be pseudo-anonymized, i.e. direct identifiers such as name will be replaced by a code in all official study documents.
- The participant is at least 18 years old
- The respondent consented (with signature or fingerprint) to participate in the study.

Investigator Name ____________________________________

Date (dd/mm/yy) __/__/__ Investigator Signature ________________________

Participant number: ___

| INFORMATION FORM FOR FORMAL MEDICINE PROVIDERS |
| --- |

You are invited to voluntarily participate in a study conducted by the Clinical Research Unit of Nanoro (CRUN), the Institute of Tropical Medicine, Antwerp in Belgium, the University of Cambridge and the University of Oxford in the United Kingdom. The study and the intervention it will evaluate are described in this information sheet. If you have any questions about the study or intervention, please don’t hesitate to ask. In addition, you are of course free to interrupt your participation in the study or intervention at any time.

Purpose and description of the study

The increase in antibiotic resistance makes drugs currently used for bacterial infections ineffective. Before developing and introducing interventions that would enable better use of antibiotics in the community and prevent infections with resistant bacteria, we should first understand where, when, how and why antibiotics are used and under what conditions and practices of hygiene and sanitation pathogens transfer are facilitate between people. This is why this study is proposed.

From a consultation of the stakeholders involved, the aim is to develop a package of interventions (one for medicines providers and one for the entire population) that responds to the risk factors of antibiotic resistance, in order to improve the quality of care and meet the perceived needs of health professionals or the population.

Interventions and surveys will be implemented in 11 communities, and surveys will also be carried out in 11 other control communities – but without interventions. These surveys are:

- **Patients visit exit interviews** will be carried out at the end of a visit to a formal medicine provider before and one year after the intervention to measure a possible change in the use of antibiotics,
- **Simulated patients visits** will be carried out before and one year after the intervention to evaluate a possible change in the quality of care: actors will present themselves as patients with 5 different clinical presentations, and healthcare will be evaluated from a check list.

You will be asked to participate in the development of interventions, and - if you are a medicine dispenser in the study area - your agreement for your patients to participate in surveys on antibiotic use. You will be then be visited by stakeholders and thus the quality of care will be evaluated.

Time required for participation

You will be asked to set aside approximately one hour for an initial interview. A second one-hour session will be planned with some participants from the first session, for feedback on the proposed interventions.

The intervention will be implemented during one year, and will be offered in the form of training, feedback sessions, clinical discussions between peers (therefore between health actors) according to identified needs.

The intervention evaluation surveys will not require dedicated time from health actors.

Voluntary participation

Your participation in this study is entirely voluntary and this choice is not documented or reported in the study materials.

Don't hesitate to ask if there is anything you don't understand or ask for explanations until you are satisfied. You are free to withdraw or stop your participation at any time.

If you wish to participate, indicate that you agree to participate in the study by signing the consent form at the end of this document.

Privacy

All information collected about you during the study will be kept strictly confidential. Your name is not recorded and any information you provide will be kept confidential. Only two researchers from the research team will have access to the data and information collected. It is possible that an excerpt from the interview will be used in the dissemination of our results, but we will ensure that any information identifying you is removed from the quote.

Under no circumstances will your identity or contact details be shared with authorities or health authorities. We guarantee that all information will be treated strictly anonymously, and your name will be replaced by a code in all official study documents.

In the event of a group discussion, we ask you to respect the other participants (their anonymity) as well as the confidential nature of the conversations that will take place. However, if you are uncomfortable participating in a group conversation/discussion, we can answer your questions to reassure you. However, if you wish, you can cancel your participation.

Once you have confirmed your participation, we would like to record the audio of our conversation for research purposes only. You are free to refuse it, however, it will help our analysis and our work. All audio-recorded information will be destroyed after the study.

Benefits

There is no direct personal benefit to you from agreeing to participate in this study. However, consultation gives you the opportunity to propose interventions that can contribute to better public health in the community. Medicine dispensers will be able to participate in educational activities or scientific support, such as training and treatment tools, in order to optimize the use of antibiotics and thus improve patient care. Those who will be in the intervention communities will receive them for one year, the others after the year of evaluation of the intervention package. There is no financial compensation for participating in this study. If you had to travel, transport costs will be reimbursed (a lump sum).

Ethics committee

This study was reviewed by the institutional review board of the Institute of Tropical Medicine, the ethics committee of the University of Antwerp, Belgium, and the National Ethics Committee for Health Research in Burkina Faso, and received a favorable recommendation.

If you have any questions or comments about the study now, during or after your participation, you can contact the study coordinator: VALIA Daniel; Phone: +226 70285553; Email: valiadaniel@yahoo.com

----------------------------------

| INFORM CONSENT FORM FOR FORMAL MEDICINE DISPENSERS |
| --- |

Name of respondent (first name + last name): __________________________________

Signature : ______________________________

If the respondent cannot read or write, a witness, independent of the research team, for example a member of the same household or a neighbor, must be present during the interview for informed consent. If at the end of the interview the person agrees to participate, the witness will sign this consent form, and the participant will record the thumbprint in place of the signature.

WITNESS

Name of witness (first name + last name) : __________________________________

Signature : _____________________________

INVESTIGATOR

- I confirm that the information sheet has been read and explained to the respondent in a language that the respondent understands well.
- I ensured that the respondent understood that he/she can freely choose to participate in the study, that he/she does not have to answer a question that he/she prefers not to answer, and that he/she can end the interview at any time.
- The respondent had the opportunity to ask questions about the study and its objectives. If the respondent had questions, I or a colleague from the research team answered the questions correctly and clearly.
- The participant was explained that their name is not recorded except on the present form, which will be kept secure, only used if the participant would prefer to cancel their participation in the study, and destroyed one year after the end of the study. The information provided will be pseudo-anonymized, i.e. direct identifiers such as name will be replaced by a code in all official study documents.
- The participant is at least 18 years old
- The respondent consented (with signature or fingerprint) to participate in the study.

Investigator Name ____________________________________

Date (dd/mm/yy) __/__/__ Investigator Signature ________________________

Participant number: ___

| INFORMATION FORM FOR THE PARTICIPATION IN THE ANTIBIOTICS USE SURVEY |
| --- |

You are invited to voluntarily participate in a study conducted by the Clinical Research Unit of Nanoro (CRUN), the Institute of Tropical Medicine, Antwerp in Belgium, the University of Cambridge and the University of Oxford in the United Kingdom, by answering a few questions, mainly about treatments used in your community. The study is described in this factsheet. If you have any questions, now or later, please ask, including about the benefits and the disadvantages of the study. In addition, you are of course free to interrupt your participation in the study at any time.

Purpose and description of the study

The purpose of this survey is to understand the use of certain medications to treat certain illnesses in your community, particularly antibiotics. The results will help improve an awareness-raising intervention for the community and health workers, which will aim to reduce the spread of diseases and improve the quality of care offered to the community.

We will ask you some questions about the medicines that have been prescribed to you, given to you, or that you have purchased, for example the dosage, the frequency of medication. You will also be asked some questions about why you sought these medicines here, from this health worker or medicine supplier.

We will not collect personal data that identifies you, such as your name or date of birth. Your name appears only on this form but not in the study data. Data without identifiers could be used in future studies on drug use.

Time required for participation

Answering the questions should take a maximum of 20 minutes.

Voluntary participation

Your participation in this study is completely voluntary, i.e. you have the right to refuse to participate, now or later, without any consequences. The choice not to participate will not be documented or reported in the study documents.

Don't hesitate to ask if there is anything you don't understand until you are satisfied. You are free to withdraw or stop your participation at any time. You are also free to choose the questions you want to answer.

If you wish to participate, we will ask you to confirm in writing that you agree to participate in the study, and you will keep a copy of this "consent" document, signed by both of us.

Privacy

All information collected during the study will be kept strictly confidential. Your name is not recorded and any information you provide will be kept confidential. Only the research team will have access to the data and information collected. We guarantee that all information will be treated strictly confidentially, and your name will be replaced by a code in all study documents.

Risks and benefits

There is no direct risk associated with this study. If any questions make you uncomfortable, please feel free at any time not to answer the question, take a break, or stop participating in this study. There is no direct individual benefit from participation in this study, no financial compensation. However, at the community level, the intervention aims to reduce antibiotic resistance and the spread of disease, and to improve patient care.

Ethics committee

This study was reviewed by the institutional review board of the Institute of Tropical Medicine, the ethics committee of the University of Antwerp, Belgium, and the National Ethics Committee for Health Research in Burkina Faso, and received a favorable recommendation.

If you have any questions or comments about the study now, during or after your participation, you can contact the study coordinator: VALIA Daniel; Phone: +226 70285553; Email: [valiadaniel@yahoo.com](mailto:valiadaniel@yahoo.com)

-------------------------

| INFORM CONSENT FORM FOR THE PARTICIPATION IN THE ANTIBIOTICS USE SURVEY |
| --- |

PARTICIPANT (adulte)

Name of respondent (first name + middle name (if available) + last name) :

__________________________________________________________________

Date (dd/mm/yy) : ___ / ___ / ___

Signature : ____________________________________________________________

If the respondent cannot read or write, a witness, independent of the research team, for example a member of the same household or a neighbor, must be present during the interview for informed consent. If at the end of the interview the person agrees to participate, the witness will sign this consent form, and the participant will record the fingerprint instead of the signature.

WITNESS

Name of witness (first name + middle name (if available) + last name) :

__________________________________________________________________

Date ddj/mm/yy) : ___ / ___ / ___

Signature : ____________________________________________________________

INVESTIGATOR

I confirm that the information sheet has been read and explained to the participant named above in a language that the respondent understands well.

I ensured that the participant understood that he/she can freely choose to participate in the study, that he/she does not have to answer a question that he/she prefers not to answer, and that he/she can end the interview at any time.

The participant had the opportunity to ask questions about the study and its objectives. If the respondent had questions, I or a colleague from the research team answered the questions correctly and clearly.

The participant was explained that their name is not recorded except on the present form, which will be kept secure, only used if the participant would prefer to cancel their participation in the study, and destroyed one year after the end of the study. The information provided would remain pseudo-anonymized, meaning that direct identifiers such as name will be replaced by a code in all official study documents.

Investigator name (first name + middle name (if available) + last name):

__________________________________________________________________

Date (dd/mm/yy) : __ / __ / __

Signature : ____________________________________________________________

| INFORM CONSENT FORM FOR ADOLESCENT PARTICIPANT IN THE ANTIBIOTICS USE SURVEY (adolescent with parent or guardian) |
| --- |

PARENT/ PARTICIPANT ADULT GUARDIAN

Name of respondent (first name + last name): __________________________________

Date (dd/mm/yy) : __ _/ ___ / ___ Signature : ______________________________

If the respondent cannot read or write, a witness, independent of the research team, for example a member of the same household or a neighbor, must be present during the interview for informed consent. If at the end of the interview the person agrees to participate, the witness will sign this consent form, and the participant will record the fingerprint instead of the signature.

WITNESS

Name of witness (first name + last name) : __________________________________

Date (dd/mm/yy) : ___ / ___ / ___ Signature : ______________________________

INVESTIGATOR

- I confirm that the information sheet has been read and explained to the adolescent participant and their parent/guardian named above in a language they understand well.
- I have ensured that the participant and his/her parent/guardian understand that they can freely choose to participate in the study, and that he/she does not have to answer a question that he/she prefers not to answer, and that he/she can end the interview at any time.
- The adolescent participant and their parent/guardian were given the opportunity to ask questions about the study and its objectives. If there were any questions, I or a colleague from the research team answered the questions correctly and clearly.
- The adolescent participant and their parent/guardian were explained that their name is not recorded except on the present form, which will be kept secure, only used if the respondent would prefer to cancel their participation in the study, and destroyed one year after the end of the study. The information provided would remain pseudo-anonymized, meaning that direct identifiers such as name will be replaced by a code in all official study documents.
- Participant age is between 14 and 17 years old.

Name of investigator: ___________________________________________________

Date (dd/mm/yy) : __ _/ ___ / ___ Signature : ________________________________

| INFORM CONSENT FORM FOR THE PARTICIPATION IN THE ANTIBIOTICS USE SURVEY  (child with parent or guardian) |
| --- |

RESPONDENT (parent or guardian of the child)

Name of respondent (first name + last name) : __________________________________

Date (dd/mm/yy) : ___ / ___ / ___ Signature : ______________________________

If the respondent cannot read or write, a witness, independent of the research team, for example a member of the same household or a neighbor, must be present during the interview for informed consent. If at the end of the interview the person agrees to participate, the witness will sign this consent form, and the participant will record the fingerprint instead of the signature.

WITNESS

Name of witness (first name + last name): __________________________________

Date (dd/mm/yy) : ___ / ___ / ___ Signature : ______________________________

INVESTIGATOR

- I confirm that the information sheet has been read and explained to the respondent named above in a language that he understands well.
- I ensured that the respondent and the child understood that he/she/they can freely choose to participate in the study, that the respondent does not have to answer a question which he/she prefers not respond, and that he/she can end the interview at any time.
- The respondent had the opportunity to ask questions about the study and its objectives. If the respondent had questions, I or a colleague from the research team answered the questions correctly and clearly.
- The respondent was explained that his name is not recorded except on the present form, which will be kept secure, only used if he would prefer to cancel his participation in the study, and destroyed one year after the end of the study. The information provided would remain pseudo-anonymized, meaning that direct identifiers such as name will be replaced by a code in all official study documents.

Name of investigator: ___________________________________________________

Date (dd/mm/yy) : ___ / ___ / ___ Signature : ________________________________

| INFORMATION FORM FOR THE SURVEY ON ASYMPTOMATIC CARRIAGE AND RESISTANT ENTEROBACTERIACEAE TRANSMISSION |
| --- |

You are invited to voluntarily participate in a study conducted by the Clinical Research Unit of Nanoro (CRUN), the Institute of Tropical Medicine, Antwerp in Belgium, the University of Cambridge and the University of Oxford in the United Kingdom, by responding for yourself, for your child or for the child for whom you are the legal guardian. At the end of the questionnaire, we will also ask you for a stool sample for laboratory analysis. The study is described in this factsheet. If you and/or your child have any questions, now or later, please ask, including advantages and disadvantages of the study.

Purpose and description of the study

Increasing antibiotic resistance makes drugs currently used to treat these infections ineffective. This means that when you or a member of your family suffer from an illness caused by resistant bacteria, the antibiotics available on the market which were once effective will no longer be able to cure you. Your treatment could therefore require hospitalization and the use of rare and very expensive antibiotics. In the stools of healthy people, there is a number of bacteria called enterobacteria. These bacteria can be transfered from one member of the community or household to another, all in good health, via hands, food, water or sharing the same toilet or latrine, without them getting sick from it. When the health of a child or an adult deteriorates, these bacteria can become opportunistic and enter the bloodstream, which can cause septicemia, a very serious illness that can lead to death. If these bacteria in the stool are resistant to antibiotics, infections in people who become ill from one of these bacteria may therefore be difficult to treat with antibiotics.

The goal of our study is to understand the extent of these resistant bacteria in your community, how often these bacteria pass from one member of a household to another, and what factors are linked to transmission. At the same time, we will implementing an awareness-raising intervention in the community and among healthcare providers, which will aim to reduce the spread of resistant bacteria. Their susceptibility testing will help use better antibiotic treatments in the event of illness caused by such bacteria. We will estimate the effect of this intervention on bacteria in the stools.

We are going to ask each member of this household to collect a stool sample to test for the bacteria that we told you about previously, in a small container that we will collect tomorrow. At the same time, we will ask each of you a few questions regarding the possession and use of antibiotics at home along with the type of antibiotics and the use of antibiotics in the past month.

Note that the presence of these bacteria does not mean that you or your child is sick. From the sample, the goal is to isolate one or more bacteria, and to investigate their characteristics, such as their resistance and their relationship to other isolated bacteria. These bacteria isolates will not have personal information on the label. The work of isolating and characterizing the bacteria will be done at Nanoro. It is possible that some bacteria that are isolated will be stored, used in future studies, or shipped from one study partner to another. If you do not agree that bacteria be stored, used in other studies, or sent, and only agree to be used to determine carriage and transmission, please let us know (and let the investigator enter it on the question sheet).

Time required for study participation

Answering the questions should take a maximum of 5 minutes per household member. For the stool sample, the interviewer will provide one sample pot per participating household member. We will return the day after consent to collect the stools.

Voluntary participation

Participation in this study by each member of the household is entirely voluntary, i.e. you have the right to refuse to participate without any consequences.

Don't hesitate to ask if there is anything you don't understand or ask for explanations until you are satisfied.

If you wish to participate, we will ask you to confirm in writing from each member of the household and from a parent or legal guardian of the children whether you agree to participate in the study, and you will keep a copy of this “consent” document, signed by both of us. For children over 14 years old, we will ask if he/she agrees to participate after receiving approval from the parent or legal guardian.

Privacy

All information collected during the study will be kept strictly confidential. Your names and children's names will not be recorded, and a code will be used in all study documents.

Risks and benefits

There is no risk for your health or that of the child by participating in this study. At the same time, there is no direct individual benefit for participating in this study, no financial compensation. However, at the community level, the intervention aims to reduce antibiotic resistance and the spread of disease. Also, systematic deworming will be offered to each member of the household every six months.

Ethics committee

This study was reviewed by the institutional review board of the Institute of Tropical Medicine, the ethics committee of the University of Antwerp, Belgium, and the National Ethics Committee for Health Research in Burkina Faso, and received a favorable recommendation.

If you have any questions or comments about the study now, during or after your participation, you can contact the study coordinator: VALIA Daniel; Phone: +226 70285553; Email: [valiadaniel@yahoo.com](mailto:valiadaniel@yahoo.com)

-------------------------

| CONSENT FORM FOR THE SURVEY ON ASYMPTOMATIC CARRIAGE AND RESISTANT ENTEROBACTERIACEAE TRANSMISSION |
| --- |

The consent form will be completed per participating household member

PARTICIPANT (if adult) or PARENT/GUARDIAN (if participant is <18 years old)

Name of respondent (first name + last name) : __________________________________

Date (dd/mm/yy) : __ / __ / __ Signature : ______________________________

If the respondent cannot read or write, a witness, independent of the research team, for example a member of the same household or a neighbor, must be present during the interview for informed consent. If at the end of the interview the person agrees to participate, the witness will sign this consent form, and the participant will record the fingerprint in place of the signature.

WITNESS

Name of witness (first name + last name) : __________________________________

Date (dd/mm/jj) : __ / __ / __ Signature : ______________________________

INVESTIGATOR

- I confirm that the information sheet has been read and explained to the respondent named above (if the patient concerned is an adolescent) and to the participant concerned in a language they understand well.
- I have ensured that the respondent (if the patient concerned is an adolescent) and the participant have understood that he/she/they can freely choose to participate in the study, that the respondent does not have to respond to a question that he/she prefers not to answer, and that he/she can end the interview at any time.
- The respondent had the opportunity to ask questions about the study and its objectives. If the respondent had questions, I or a colleague from the research team answered the questions correctly and clearly.
- The respondent was explained that their name is not recorded except on the present form, which will be kept secure, only used if the respondent would prefer to cancel their participation in the study, and destroyed one year after the end of the study. The information provided would remain pseudo-anonymized, meaning that direct identifiers such as name will be replaced by a code in all official study documents.

Investigator Name: ___________________________________________________

| INFORMATION FORM FOR ADOLESCENT STUDY PARTICIPANTS |
| --- |

You are invited to voluntarily participate in a study conducted by the Clinical Research Unit of Nanoro (CRUN), the Institute of Tropical Medicine, Antwerp in Belgium, the University of Cambridge and the University of Oxford in the United Kingdom. The study is described in this information sheet. If you have any questions about the study or intervention, please don’t hesitate to ask. In addition, you are of course free to interrupt your participation in the study or intervention at any time.

Purpose and description of the study

The purpose of this study is to understand the use of certain medications to treat certain illnesses in your community, particularly antibiotics. Increasingly, medications (antibiotics) currently used to treat illnesses are no longer effective. To try to solve this problem, we will develop and introduce a set of activities that will allow better use of antibiotics in your community while preventing certain infectious diseases. Thus, we should understand where, when, how and why antibiotics are used and what facilitates the transmission of diseases from one person to another. This is why we organize interviews, discussions and surveys with people who give or receive care. The results of this study will help evaluate an information and training campaign for health workers, in order to improve the quality of care in the community and to ensure that the drugs (antibiotics) that really respond to the needs of care are now well used to combat certain diseases that you encounter.

We will not collect personal data that identifies you, such as your name or date of birth. The name of your parent or guardian appears only on this form and not in the study data.

We have contacted your family because we want to include young people, their thoughts and ideas in our research. We are asking young people aged 14 to 17 to participate in our research. We will also ask others, such as healthcare professionals and community members, about antibiotics and maintaining health in the community. When the intervention activities are developed, we will implement them in 11 communities and see if there are any changes afterwards. We will examine the use of antibiotics and changes in hygiene practices. We will examine them before the intervention and one year after.

If you agree to participate in this study and at least one of your parents gives permission, you will be encouraged to think and talk about the use of antibiotics in your communities and families, as well as hygiene at home, at school and in the community.

Time required for participation

You will be asked to set aside approximately one hour for the interview/discussion.

Voluntary participation

Your participation in this study is completely voluntary, i.e. you have the right to refuse to participate, now or later (at the time of the home visits), without any consequences. The choice not to participate will not be documented or reported in the study documents.

Don't hesitate to ask if there is anything you don't understand until you are satisfied. You are free to withdraw or stop your participation at any time. You are also free to choose the questions you want to answer.

If you wish to participate, we will ask for your (adolescent's) agreement, and for your parent/guardian to sign or fingerprint acceptance of participation in the study, and you will keep a copy of this “consent” document, signed by me and the parent/guardian.

Privacy

All information collected during the study will be kept strictly confidential. Your name and your child's name are not recorded and any information you provide will be kept confidential. Only the research team will have access to the data and information collected. The contact (phone number) of the parent will be kept until the follow-up call, and not kept afterwards. We guarantee that all information will be treated strictly confidentially, and your name will be replaced by a code in all study documents.

Benefits

Although you will not receive any direct benefit from your participation, others may benefit from the knowledge gained from this study. There is no compensation for participating in this study. If you had to travel, transport costs will be reimbursed (a lump sum).

Ethics committee

This study was reviewed by the institutional review board of the Institute of Tropical Medicine, the ethics committee of the University of Antwerp, Belgium, and the National Ethics Committee for Health Research in Burkina Faso, and received a favorable recommendation.

If you have any questions or comments about the study now, during or after your participation, you can contact the study coordinator: VALIA Daniel; Phone: +226 70285553; Email: [valiadaniel@yahoo.com](mailto:valiadaniel@yahoo.com)

------------------------

| INFORM CONSENT FORM FOR ADOLESCENT STUDY PARTICIPANTS |
| --- |

PARENT OR GUARDIAN of the participating adolescent

Name of parent/guardian(first name + last name): __________________________________

Date (dd/mm/yy) : __ / __ / __ Signature : ______________________________

If the respondent cannot read or write, a witness, independent of the research team, for example a member of the same household or a neighbor, must be present during the interview for informed consent. If at the end of the interview the person agrees to participate, the witness will sign this consent form, and the participant will record the fingerprint instead of the signature.

WITNESS

Name of witness (first name + last name) : __________________________________

Date (dd/mm/yy) : __ / __ / __ Signature : ______________________________

VERBAL ASSENT BY THE PARTICIPANT (to be completed by the researcher for each adolescent participant)

- I confirm that the information sheet has been read and explained to the adolescent participant and their parent/guardian named above in a language they understand well.
- I have ensured that the patient and his/her parent/guardian understand that they can freely choose to participate in the study, and that he/she does not have to answer a question that he/she prefers not to answer, and that he/she can end the interview at any time.
- The adolescent participant and their parent/guardian were given the opportunity to ask questions about the study and its objectives. If there were any questions, I or a colleague from the research team answered the questions correctly and clearly.
- The adolescent participant and their parent/guardian were explained that their name is not recorded except on the present form, which will be kept secure, only used if the respondent would prefer to cancel their participation in the study, and destroyed one year after the end of the study. The information provided would remain pseudo-anonymized, meaning that direct identifiers such as name will be replaced by a code in all official study documents.
- The age of the participant is between 14 and 17 years old.

Investigator Name ____________________________________

Date (jj/mm/aa) __/__/__ Investigator Signature ________________________

Participant number: ___
