# Supplementary material for: Evaluating the effect of a behavioural intervention bundle on antibiotic use, quality of care, and household transmission of resistant Enterobacteriaceae in intervention versus control clusters in rural Burkina Faso and DR Congo (CABU-EICO)
Source: Trials. 2024 Jan 27;25:91. doi: 10.1186/s13063-023-07856-2 (PMC10821568; doi:10.1186/s13063-023-07856-2)
Supplement: Supplementary file 3 — Additional file 3. Biological specimens statement. [file 13063_2023_7856_MOESM3_ESM.docx]

Biological specimen statement.

Objective: stools will be collected to fenotypically and genotypically determine AMR

**Background: The CABU-EICO trial assess the effect of a behavioural intervention on community antibiotic use and related behaviours, to reduce the AMR risks. Analysis of stools before, during and after the intervention, in both humans and rodents, enables us to estimate long-term effects on community AMR.**

**Methods:** Stool samples will be cultured on selective CHROMagar™ plates and species identified. If *E. coli* and/or *Salmonella* isolates are detected, they will be typed and antibiotic susceptibility testing done according to CLSI guidelines for pathogen-antibiotic indicators and for ESBL, if the minimal inhibitory concentration for ceftriaxone or ceftazidime is above 1 mg/L.

A selection of ESBL-producing *E. coli* isolates will be sequenced. DNA will be extracted and prepared for whole genome sequencing (WGS) using Illumina technology available through the Cancer Research UK facility. The WGS data will be bioinformatically assessed for quality and analysed to confirm species and sequence type per isolates. Furthermore, the genetic content of AMR genes and the presence of plasmid replicons will be determined. In-depth comparative genomics approach will be used to assess relatedness between isolates through reconstruction of the maximum likelihood phylogenies per species/sequence type, with their representative metadata including AMR data.
